# Clinical Symptom Differences Between Mild and Severe COVID-19 Patients in China: A Meta-Analysis

**DOI:** 10.3389/fpubh.2020.561264

**Published:** 2021-01-14

**Authors:** Xiaobo He, Xiao Cheng, Xudong Feng, Hong Wan, Sihan Chen, Maoming Xiong

**Affiliations:** ^1^Department of General Surgery, The First Affiliated Hospital of Anhui Medical University, Hefei, China; ^2^Department of Pathology, Anhui Medical University, Hefei, China; ^3^Zhejiang University, Hangzhou, China

**Keywords:** COVID-19, clinical features, differences, risk factor, meta-analysis

## Abstract

**Objective:** The prognosis of mild and severe patients has prominent differences during the prevalence of COVID-19, and it will be significant to identify patients' potential risk of progressing to severe cases according to their first clinical presentations. Therefore, we aim to review the clinical symptoms of the COVID-19 epidemic systematically.

**Methods:**We searched PubMed, Embase, Web of Science, and CNKI (Chinese Database) for studies about the clinical features of COVID-19 in China from March 18 to April 18. Then we used REVMAN to conduct a meta-analysis.

**Results:** After screening, 20 articles including 3,326 COVID-19 confirmed cases were selected from 142 articles we retrieved at the beginning of our research. We divided all the cases into a severe group (including severe and critically severe patients) and a mild group according to the “Diagnosis and Treatment Protocol for Novel Coronavirus Infection-Induced Pneumonia” version 4 (trial). Of all the initial symptoms (including fever, cough, abdominal pain, anorexia, chest tightness, diarrhea, dyspnea, expectoration, fatigue, headache, hemoptysis, myalgia, nausea or vomiting, and pharyngalgia) we studied, we found that cough (odds ratio [OR] = 1.4, 95% confidence interval [CI]: 1.2–1.7; *p* < 0.001), fever (OR = 1.5, 95% CI: 1.2–1.9; *p* < 0.001), dyspnea (OR = 6.2, 95% CI: 3.6–10.6; *p* < 0.001), diarrhea (OR = 2.6, 95% CI: 1.3–4.9; *p* < 0.001), fatigue (OR = 2.1, 95% CI: 1.3–3.3; *p* < 0.01), expectoration (OR = 1.7, 95% CI: 1.2–2.6; *p* < 0.01), myalgia (OR = 1.6, 95% CI: 0.8–3.1; *p* < 0.001), hemoptysis (OR = 4.0, 95% CI: 1.5–11.3; *p* < 0.001), abdominal pain (OR = 7.5, 95% CI: 2.4–23.4; *p* < 0.001), and anorexia (OR = 2.8, 95% CI: 1.5–5.1; *p* < 0.001) had a different distribution in two groups and were statistically significant (*p* < 0.05).

**Conclusion:**COVID-19 patients whose initial manifestation is dyspnea, hemoptysis, anorexia, diarrhea, or fatigue, especially abdominal pain should be closely monitored to prevent disease deterioration.

## Introduction

An outbreak of a novel coronavirus-induced pneumonia occurred in Wuhan in December 2019, with the characteristics of being occult and infectious, with an incubation period of 3–7 days, usually no more than 14 days (1). On February 11, 2020, the World Health Organization (WHO) officially named the epidemic disease caused by the new coronavirus as coronavirus 2019 (coronavirus disease 2019) or COVID-19 (2). According to a study, COVID-19 can be transmitted between humans, and it has a significantly high RO (basic reproduction number, an essential index to evaluate the ability of a virus to spread), which was 3.77 times higher than that of MERS-CoV (3). An official report announced that the virus might have the feature of aerosol transmission. The virus can infect a human in a relatively closed space exposed to a high concentration of aerosols for a long time (4, 5). In the early stage of COVID-19, major symptoms were fever, dry cough, fatigue, and then breath difficulties, and severe cases can deteriorate to acute respiratory distress syndrome (ARDS) or septic shock, even death. On April 20, 2020, cumulative confirmed cases have reached 84,278 in China according to a report of WHO (6).

It is now universally acknowledged that severe COVID-19 cases have higher mortality than mild cases because severe cases are more likely to suffer ARDS, septic shock, or metabolic acidosis (7). So it is necessary to distinguish between severe and mild patients at an early stage. According to a report by Yong Gao (8), the levels of IL-6 and d-dimer can be measured to estimate the severity of COVID-19 and help to diagnose severe COVID-19 patients earlier. Also, Fei Zhou et al. (9) found that risk factors for death of adult patients with COVID-19 were a higher Sequential Organ Failure Assessment score, older age, and elevated d-dimer at admission. However, for countries or regions with poor health conditions, these methods may not be applicable. Our research aims to distinguish between severe and mild cases with COVID-19 at an early stage by analyzing initial clinical symptoms at admission, so it is easier to manipulate. We focused on 14 initial clinical presentations that most commonly occurred in COVID-19 patients and tried to determine the differences between mild and severe COVID-19 patients. It may help enable the implementation of effective interventions and likely lower the mortality of COVID-19 patients.

At present, over 200 countries are involved in this epidemic, and pneumonia induced by COVID-19 has become an enormous threat to global public health. Many cases emerged inside and outside China over the past month (2, 10–13). Although there have been many studies on clinical case analysis, the limited number of cases in each study may lead to different results and more significant bias. Therefore, our study involved over 3,000 confirmed cases to reveal the clinical symptoms of COVID-19 patients and provide help for clinical prevention and control of the epidemic disease.

## Methods

### Search Strategy and Inclusion Criteria

We retrieved four databases, PubMed (https://pubmed.ncbi.nlm.nih.gov/), Embase (https://www.embase.com/), Web of Science (http://isiknowledge.com/), and CNKI (https://www.cnki.net/), to acquire case analysis studies on coronavirus disease 2019. Articles published between March 18, 2020, and April 18, 2020 were included. The search terms we used were as follows: (“SARS-CoV-2” or “nCoV” or “COVID-19,” or “coronavirus”) AND (“clinical feature” or “clinical characteristic” or “clinical symptom”). We evaluated all the search results according to the Preferred Reporting Items for Systematic Reviews and Meta-Analyses (PRISMA) statement. Original articles related to COVID-19 patients in China without restriction on study design or study type were included. Studies on pregnant and infant patients, without reliable clinical data, and outside China were excluded.

### Data Extraction and Quality Assessment

Information, including the first author, initial symptoms researched, and a sample size of severe and mild groups, was extracted from each study, and a Microsoft Excel database was used to record the details. Two independent reviewers (Xiaobo He and Xudong Feng) conducted the selection and assessment of article quality. Any disagreement was solved by consulting a professional investigator (Maoming Xiong).

### Bias Risk Assessment

We used MINORS (Table 1) to assess the bias risk of included studies.

**Table 1:** Bias risk assessment.

**Study**	**①**	**②**	**③**	**④**	**⑤**	**⑥**	**⑦**	**⑧**	**⑨**	**⑩**	**⑪**	**⑫**	**Score**
Cai Q.	2	2	2	2	2	1	2	0	2	2	2	2	21
Dong L.	2	2	2	2	2	1	1	0	2	2	2	2	20
Fang X.	2	2	2	2	2	0	0	0	2	2	2	2	18
Guan W.	2	2	2	2	2	0	1	0	2	2	2	2	19
Huang C.	2	2	2	2	2	0	1	0	2	2	2	2	19
Li D.	2	2	2	2	2	1	1	0	2	2	2	2	20
Li K.	2	2	2	2	2	1	2	0	2	2	2	2	21
Li Y.	2	2	2	2	2	1	1	0	2	2	2	2	20
Liu K.	2	2	2	2	2	1	2	0	2	2	2	2	21
Liu W.	2	2	2	2	2	0	2	0	2	2	2	2	20
Lu Z.	2	2	2	2	2	0	0	0	2	2	2	2	18
Wan Q.	2	2	2	2	2	0	1	0	2	2	2	2	19
Wan S.	2	2	2	2	2	1	2	0	2	2	2	2	21
Wang D.	2	2	2	2	2	0	1	0	2	2	2	2	19
Wu C.	2	2	2	2	2	1	1	0	2	2	2	2	20
Xiang T.	2	2	2	2	2	1	0	0	2	2	2	2	19
Yuan J.	2	2	2	2	2	1	1	0	2	2	2	2	20
Zhang J.	2	2	2	2	2	0	1	0	2	2	2	2	19
Zhang W.	2	2	2	2	2	1	2	0	2	2	2	2	21
Zheng F.	2	2	2	2	2	0	2	0	2	2	2	2	20

* ① A clearly stated aim. Inclusion of consecutive patients. ② Prospective collection of data. ③ Endpoints appropriate to the aim of the study. ④ Unbiased assessment of the study endpoint. ⑤ Follow-up period appropriate to the aim of the study. ⑥ Loss to follow up less than 5%. ⑦ Prospective calculation of the study size. ⑧ Appropriate selection of control group. ⑨ Synchronization of control group. ⑩ Baseline comparable between groups. ⑪ Appropriately statistical analysis. ⑫ The global ideal score being 24 for comparative studies*.

### Data Analysis

We performed statistical analysis with REVMAN (Review Manager version 5.3, The Nordic Cochrane Center, The Cochrane Collaboration, Copenhagen, 2014). Crude odds ratios (ORs) and 95% confidence intervals (CIs) were used to estimate the strength of the association between clinical features and COVID-19 (*p* < 0.05 was considered statistically significant). The *I*^2^ index, which indicates the percentage of the total variation across studies, was used to assess statistical heterogeneity (14). When *I*^2^ < 50%, a fixed-effects model was used to determine OR, while when *I*^2^ > 50%, a random-effects model was selected. We used Begg's rank correlation test and Egger's weighted regression method to assess potential publication bias, with *p* < 0.05 indicating statistically significant publication bias (15).

## Results

### Study Characteristics

**Figure 1:**
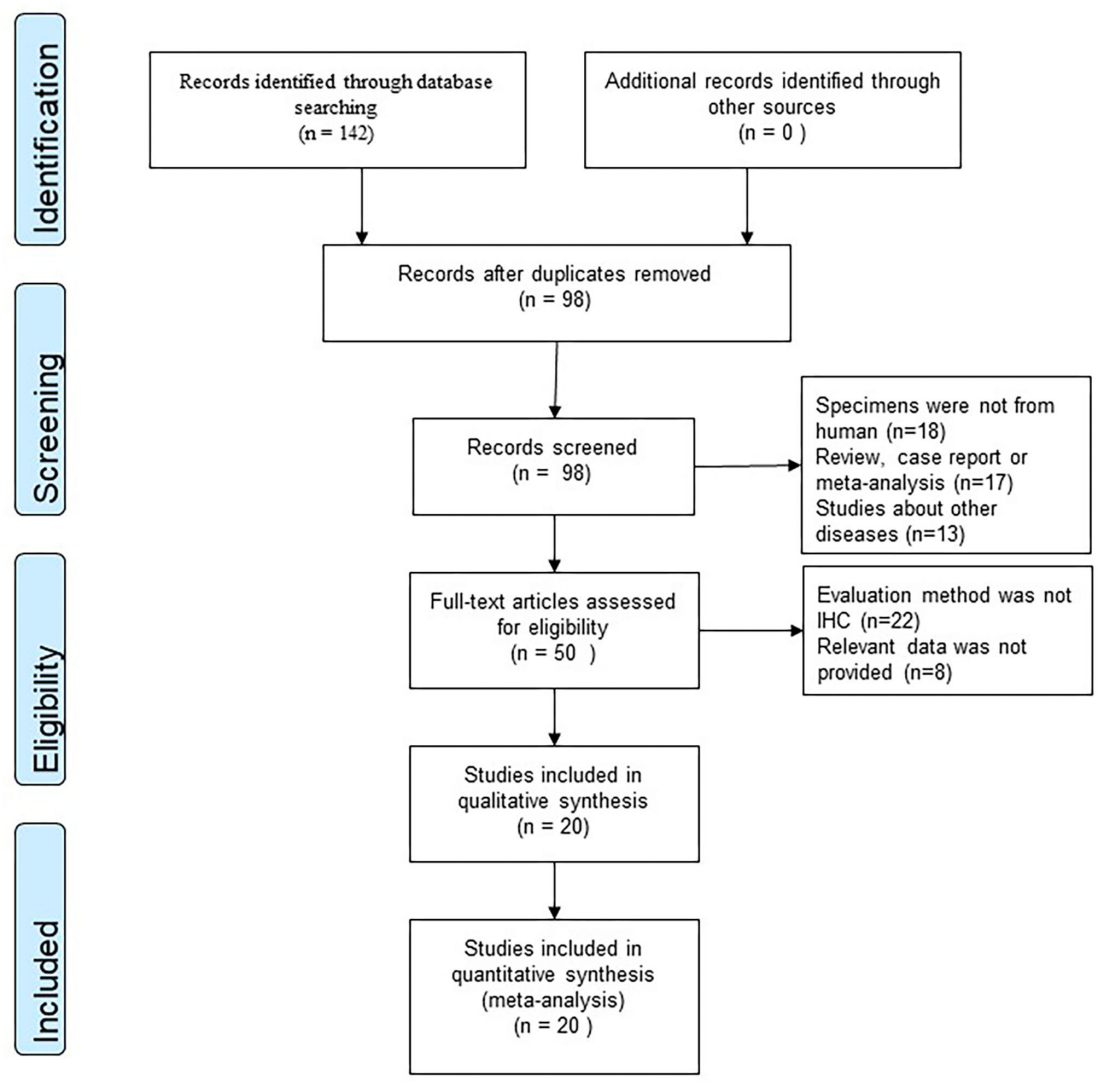
Flow diagram of the study selection procedure of the meta-analysis.

**Figure 2:**
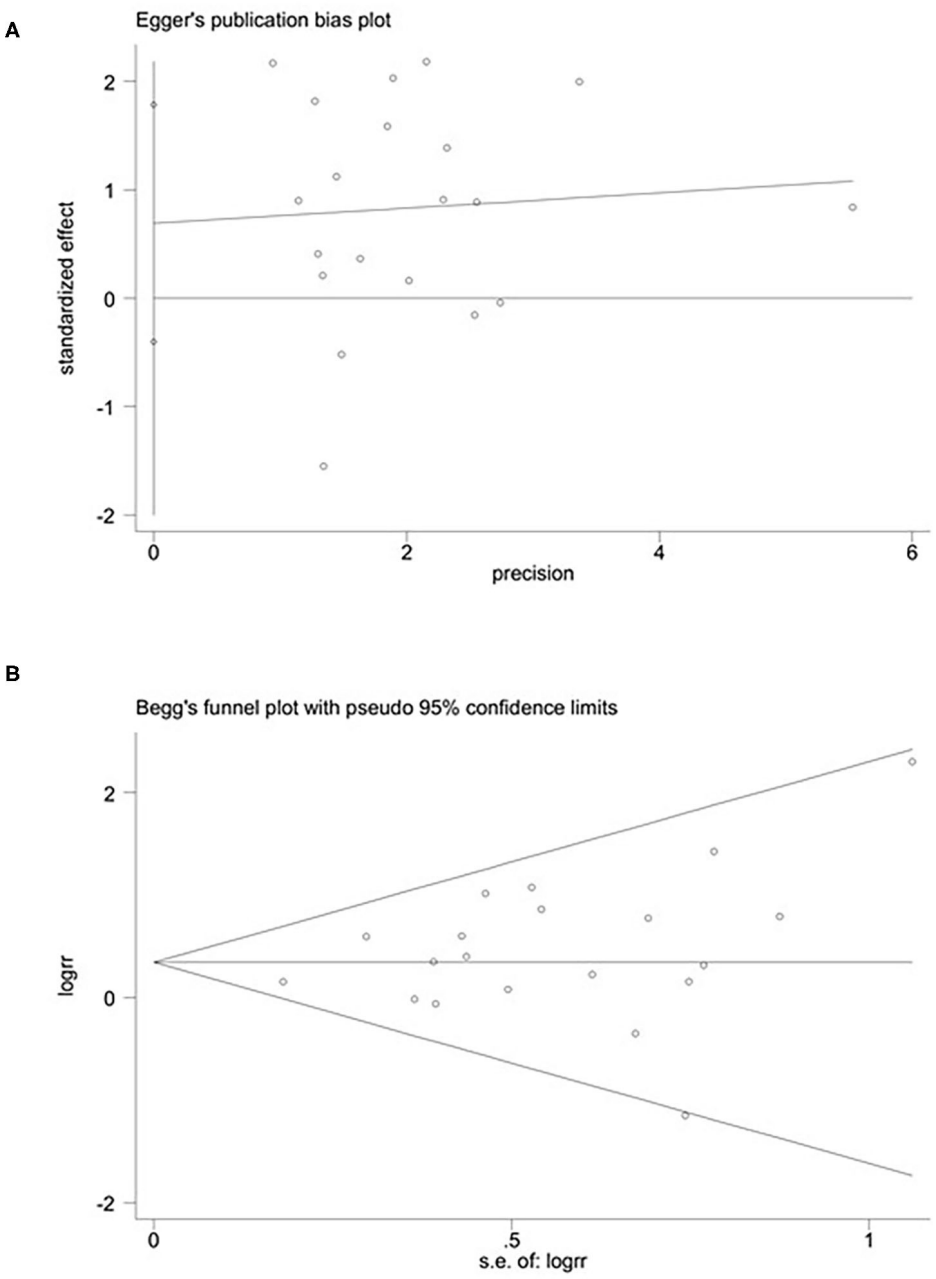
Publication bias: Begg's **(A)** (*p* > 0.05) and Egger's **(B)** (*p* > 0.05) funnel plots for possible publication bias in the current study. No publication bias was found, indicating credible results.

**Figure 3:**
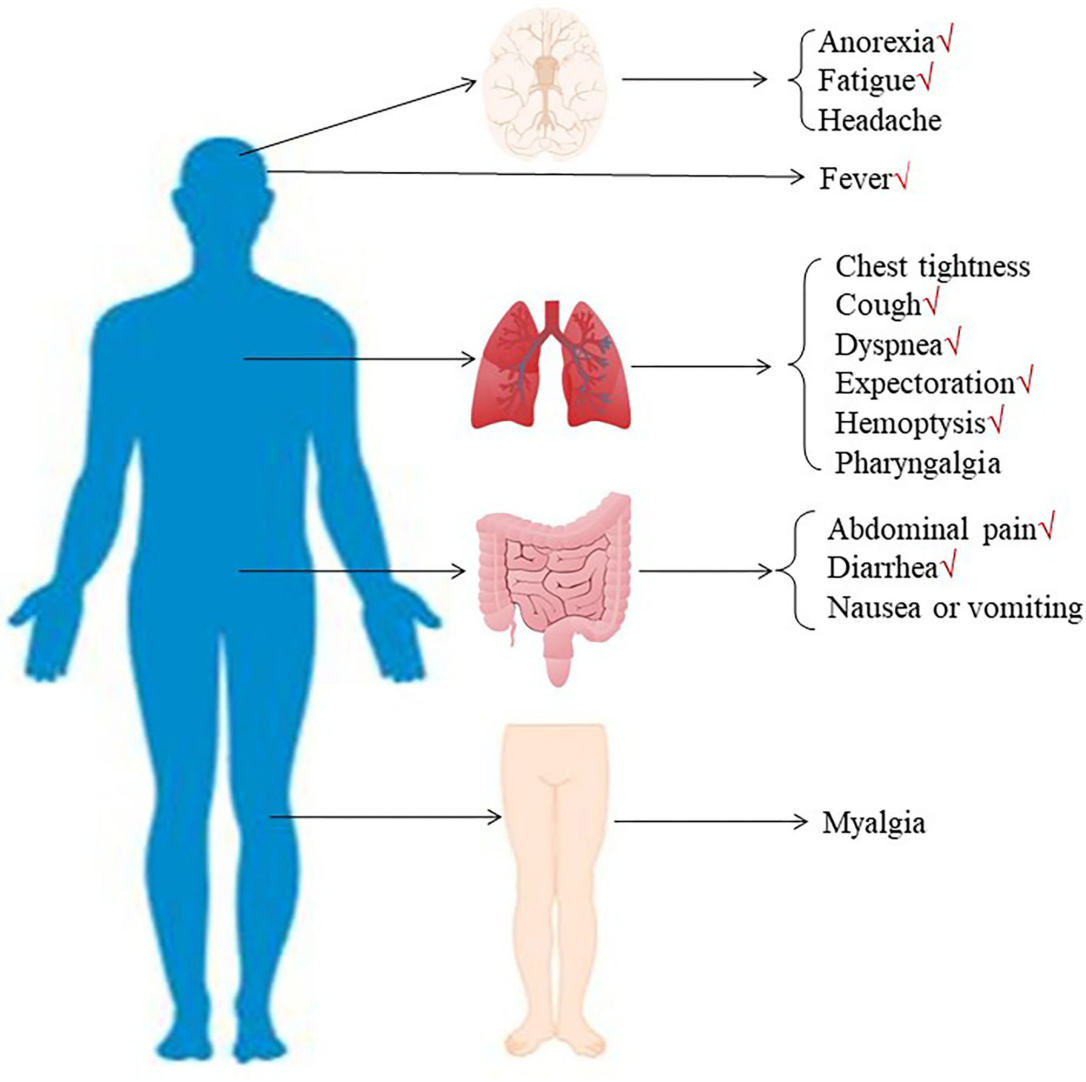
The initial symptoms were divided into three types: respiratory symptoms (including fever, cough, dyspnea, expectoration, hemoptysis, chest tightness, and pharyngalgia), digestive symptoms (including abdominal pain, diarrhea, and nausea or vomiting), and neurological symptoms (including anorexia, fatigue, myalgia, and headache).

According to our research method, we collected 142 studies from four databases (PubMed, EMBASE, Web of Science, and CNKI). Figure 1 shows the flow chart of selection, and no publication bias existed (Figure 2). Details were as follows: 98 articles were further browsed after duplicates were removed. Of these studies, those which were reviews, case reports, or meta-analyses (17 articles); related to other diseases (13 articles); or not about humans (18 articles) were excluded. Then we conducted a qualification assessment of the remaining 50 articles. Twenty-two articles were excluded for not using IHC as an evaluation method, and eight articles were excluded for lacking relevant data. Finally, 20 studies were analyzed. We extracted and analyzed 14 symptoms (including fever, cough, dyspnea, expectoration, hemoptysis, abdominal pain, diarrhea, chest tightness, headache, myalgia, nausea or vomiting, pharyngalgia, anorexia, and fatigue) with REVMAN. For research purposes, we divided all the initial symptoms into four types: fever, respiratory symptoms (including cough, dyspnea, expectoration, hemoptysis, chest tightness, and pharyngalgia), digestive symptoms (including abdominal pain, diarrhea, and nausea or vomiting), and neurological symptoms (including anorexia, fatigue, myalgia, and headache). Details of patients' clinical features and meta-analysis outcomes are shown in Tables 2, 3, respectively. The initial symptoms we studied in this research are shown in Figure 3, and the indexes marked with a symbol (√) are statistically significant.

**Table 2:** Clinical characteristics of the include studies on COVID-19.

**Study**	**Abdominal pain**		**Anorexia**		**Chest tightness**		**Cough**		**Diarrhea**		**Dyspnea**		**Expectoration**		**Fatigue**		**Headache**		**Hemoptysis**		**Myalgia**		**Nausea or vomiting**		**Pharyngalgia**	
	**S/T**	**N/T**	**S/T**	**N/T**	**S/T**	**N/T**	**S/T**	**N/T**	**S/T**	**N/T**	**S/T**	**N/T**	**S/T**	**N/T**	**S/T**	**N/T**	**S/T**	**N/T**	**S/T**	**N/T**	**S/T**	**N/T**	**S/T**	**N/T**	**S/T**	**N/T**
Cai Q.	–	–	–	–	–	–	27/58	78/240	4/58	5/240		–	–	–	3/58	10/240	0/58	5/240	–	–	–	–	–	–	0/58	2/240
Dong L.	–	–	–	–	–	–	7/14	20/45	–	–	–	–	2/14	10/45	6/14	2/45	2/14	3/45	–	–	4/14	4/45	–	–	2/14	3/45
Fang X.	–	–	–	–	–	–	14/24	31/55	3/24	1/55	4/24	5/55	3/24	7/55	–	–	–	–	–	–	–	–	–	–	2/24	1/55
Guan W.	–	–	–	–	–	–	122/173	623/926	10/173	32/926	65/173	140/926	61/173	309/926	69/173	350/926	26/173	124/926	4/173	6/926	30/173	134/926	12/173	43/926	23/173	130/926
Huang C.	–	–	–	–	–	–	11/13	20/28	0/13	1/25	12/13	10/27	5/13	6/26	–	–	0/13	3/25	1/13	1/26	–	–	–	–	–	–
Li D.	–	–	–	–	–	–	14/17	43/63	4/17	9/63	15/17	4/63	14/17	43/63	10/17	24/63	3/17	9/63	–	–	3/17	19/63	–	–	–	–
Li K.	–	–	–	–	–	–	24/25	41/58	–	–	7/25	2/58	9/25	6/58	–	–	3/25	6/58	–	–	5/25	10/58	–	–	2/25	4/58
Li Y.	–	–	–	–	–	–	18/27	19/22	27/27	4/22	–	–	–	–	24/27	8/22	–	–	–	–	–	–	–	–	–	–
Liu K.	–	–	–	–	–	–	18/21	36/43	–	–	–	–	14/21	22/43	17/21	31/43	–	–	–	–	–	–	–	–	–	–
Liu W.	–	–	–	–	–	–	4/11	30/67	–	–	6/11	14/67	–	–	–	–	–	–	–	–	–	–	–	–	–	–
Lu Z.	–	–	–	–	–	–	16/34	22/67	–	–	–	–	7/34	20/67	–	–	2/34	7/67	–	–	–	–	–	–	–	–
Wan Q.	–	–	–	–	–	–	16/21	76/132	–	–	8/21	23/132	13/21	39/132	12/21	25/132	–	–	–	–	9/21	9/132	–	–	–	–
Wan S.	–	–	–	–	3/40	9/95	35/40	67/96	13/40	5/95	18/40	0/95	7/40	5/95	–	–	11/40	23/95	3/40	1/95	–	–	–	–	0/40	24/95
Wang D.	3/36	0/102	24/36	31/102	–	–	21/36	61/102	6/36	8/102	23/36	20/102	8/36	29/102	29/36	67/102	3/36	6/102	–	–	12/36	36/102	4/36	10/102	12/36	12/102
Wu C.	–	–	–	–	–	–	68/84	95/117	–	–	50/84	30/117	–	–	–	–	–	–	–	–	–	–	–	–	–	–
Xiang T.	–	–	–	–	0/9	4/40	6/9	13/40	0/9	2/40	–	–	4/9	3/40	3/9	6/40	–	–	–	–	–	–	–	–	0/9	7/40
Yuan J.	–	–	8/57	9/82	–	–	18/31	95/192	0/31	12/192	15/31	0/192	7/31	20/192	–	–	1/31	10/192	–	–	–	–	–	–	–	–
Zhang J.	6/57	2/82	–	–	–	–	45/53	45/67	9/57	9/82	24/53	20/67	–	–	39/53	51/67	–	–	–	–	–	–	5/57	19/82	–	–
Zhang W.	3/9	3/56	0/9	1/56	–	–	3/9	15/56	3/9	3/56	–	–	1/9	5/56	0/9	8/56	0/9	2/56	–	–	–	–	0/9	1/56	0/9	3/56
Zheng F.	–	–	–	–	–	–	21/30	80/131	1/30	16/131	9/30	14/131	–	–	15/30	49/131	4/30	8/131	–	–	4/30	14/131	0/30	6/131	–	–

*S/T, severe cases/total cases; N/T, non-severe cases/total cases*.

**Table 3:** Meta-analysis outcomes of clinical features.

**Clinical features**	**Number of studies**	**Number of patients**	**Heterogeneity**		**OR (95%CI)**	***P-*value**
			***I*^**2**^(%)**	***p*-value**		
Abdominal pain	3	342	0	0.67	7.5 (2.4, 23.4)	0.0005
Anorexia	3	342	43	0.17	2.8 (1.5, 5.1)	0.0009
Chest tightness	2	184	0	0.72	0.7 (0.2, 2.4)	0.55
Cough	20	3,267	8	0.36	1.4 (1.2, 1.7)	0.0002
Diarrhea	13	2,552	56	0.007	2.3 (1.3, 4.9)	0.005
Dyspnea	13	2,590	72	< 0.00001	6.2 (3.6, 10.6)	< 0.00001
Expectoration	14	2,367	51	0.01	1.7 (1.2, 2.6)	0.009
Fatigue	13	2,436	62	0.002	2.1 (1.3, 3.3)	0.002
Fever	–	–	45	0.02	1.5 (1.2, 1.9)	0.0005
Headache	12	2,480	0	0.94	1.1 (0.8, 1.6)	0.48
Hemoptysis	3	1,273	0	0.77	4.0 (1.5, 11.3)	0.008
Myalgia	8	1,773	67	0.006	1.6 (0.8, 3.1)	0.15
Nausea or vomiting	5	1,602	44	0.9	0.9 (0.6, 1.5)	0.66
Pharyngalgia	9	2,005	54	0.03	1.2 (0.5, 2.7)	0.69

### Associations Between Fever and Severe Cases

**Figure 4:**
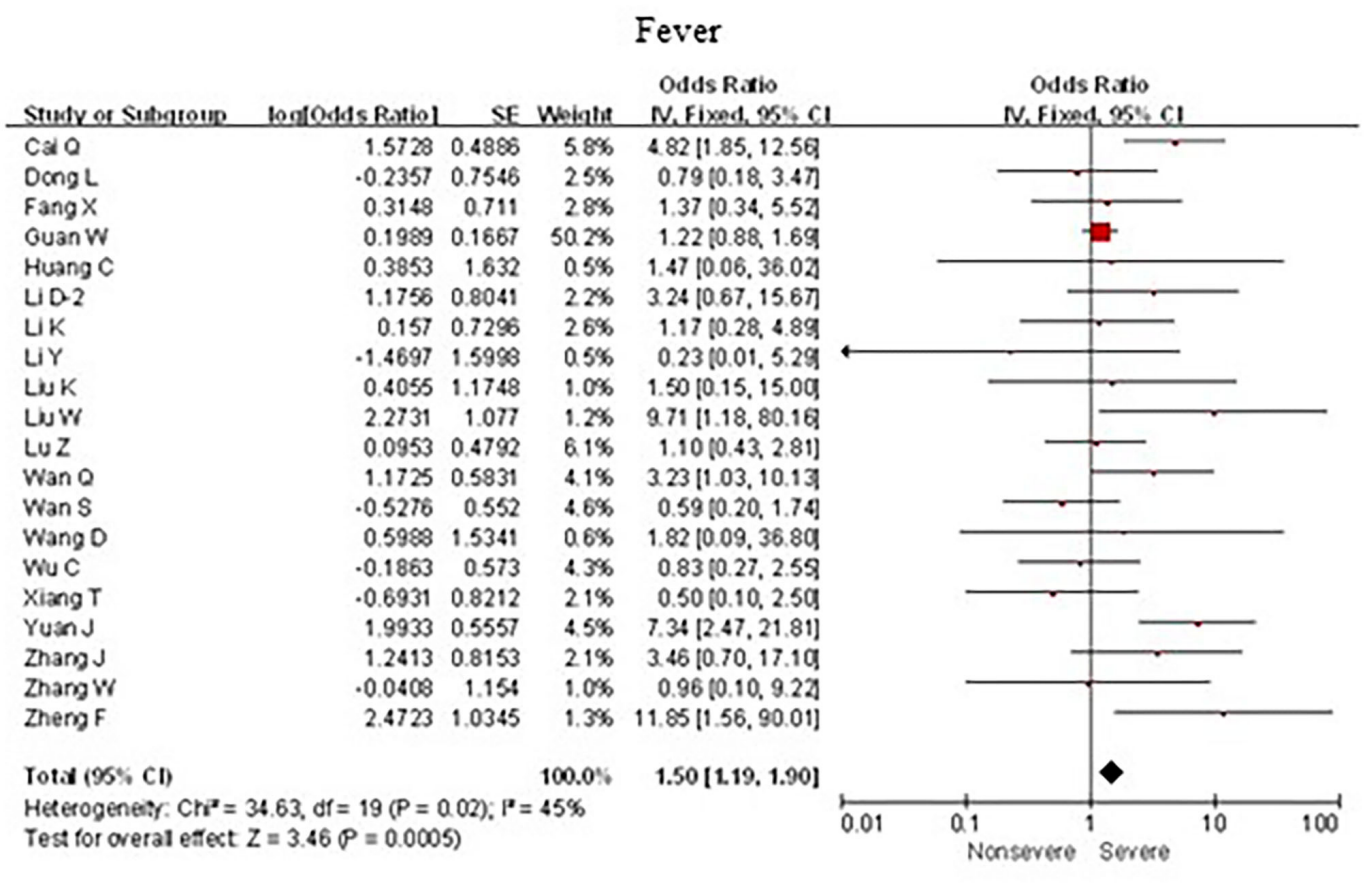
Forest plots of odds ratios (ORs) showed the associations between fever and disease progression of COVID-19 pneumonia.

As shown in Figure 4, meta-analysis results revealed that fever (OR = 1.5, 95% CI: 1.2–1.9; *p* < 0.001) frequently occurred in patients with severe COVID-19 pneumonia compared with the mild cases.

### Associations Between Respiratory Symptoms and Severe Cases

**Figure 5:**
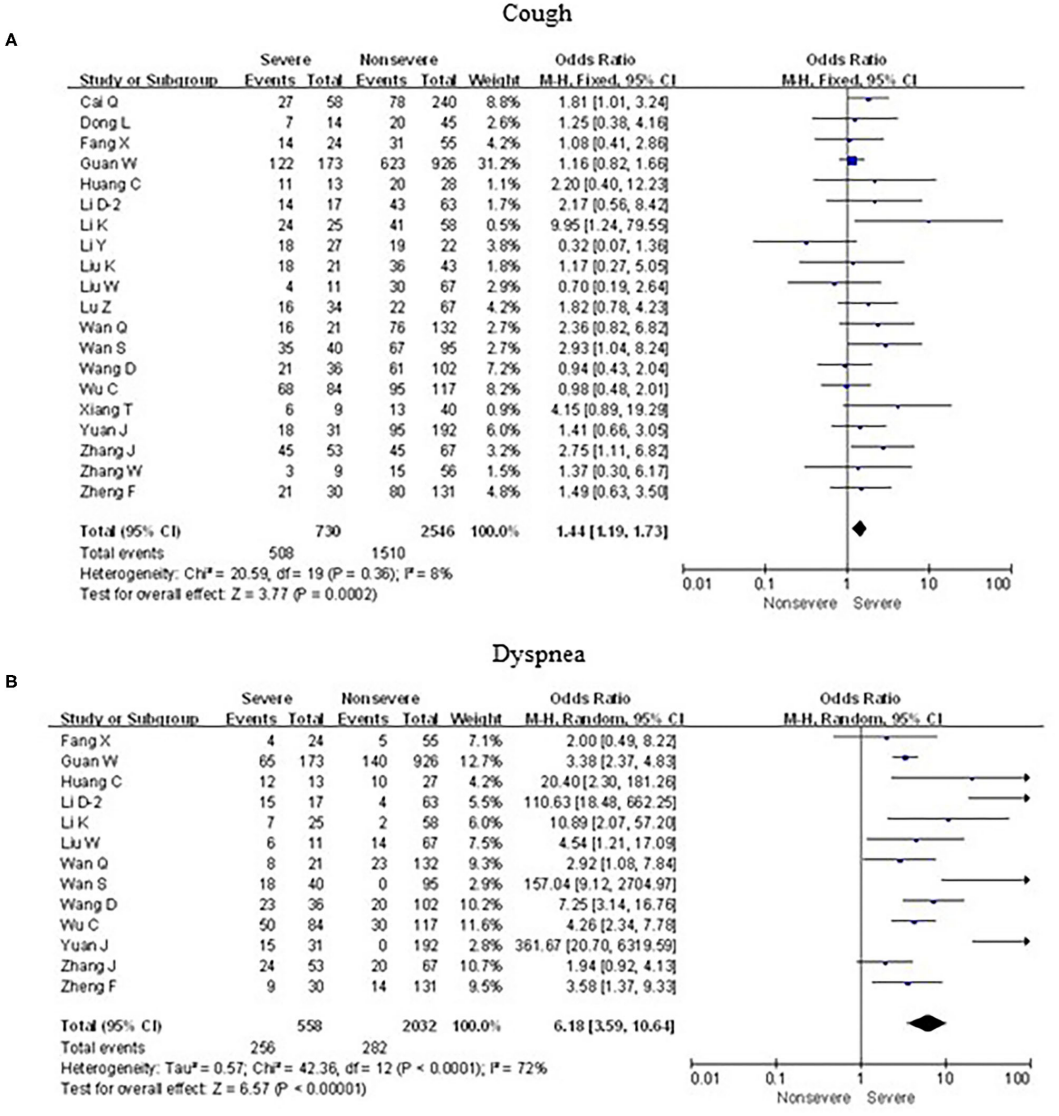
Forest plots of odds ratios (ORs) showed the associations between respiratory symptoms, including cough **(A)** as well as dyspnea **(B)**, and disease progression of COVID-19 pneumonia.

**Figure 6:**
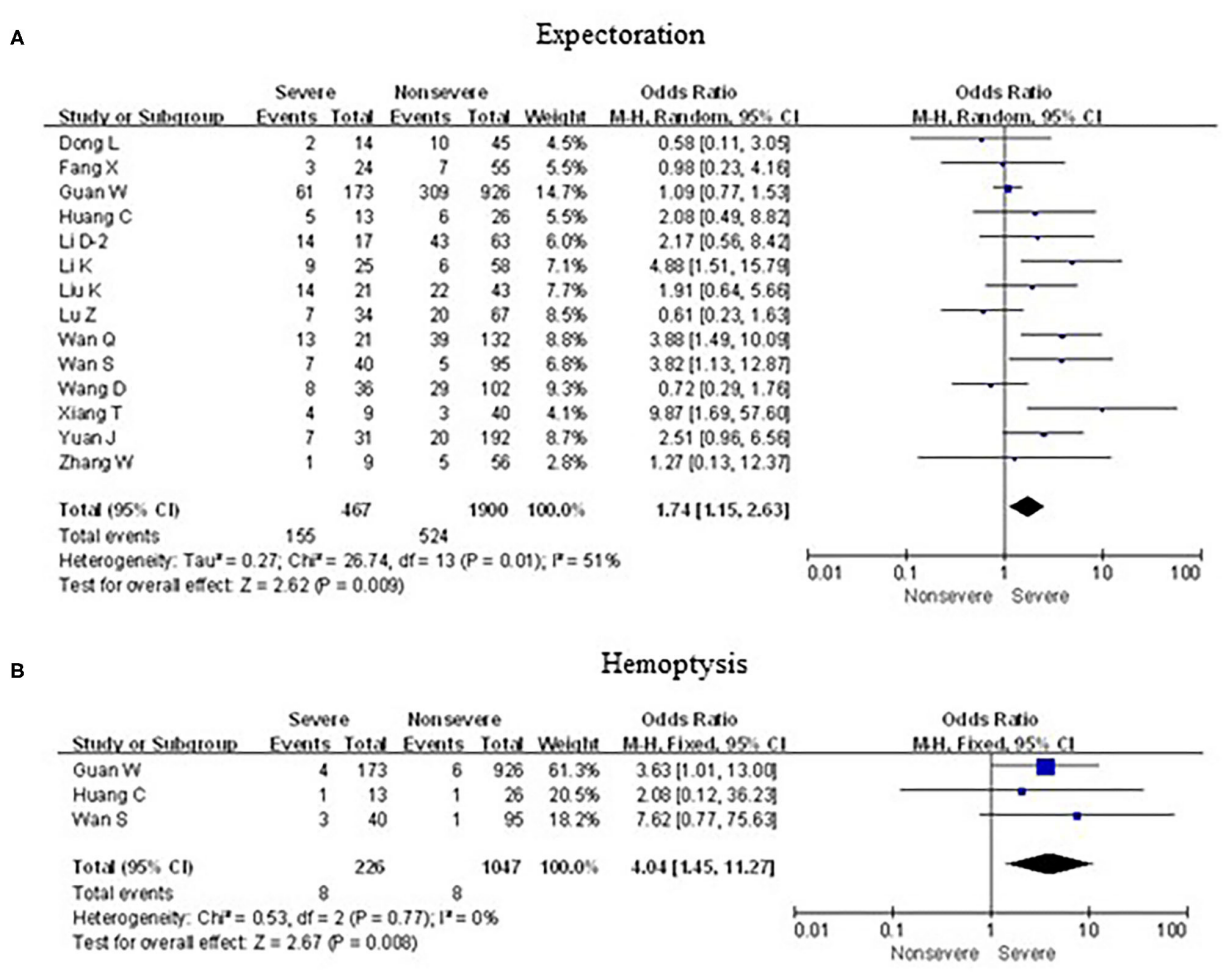
Forest plots of odds ratios (ORs) showed the associations between respiratory symptoms, including expectoration **(A)** as well as hemoptysis **(B)**, and disease progression of COVID-19 pneumonia.

As shown in Figures 5, 6, meta-analysis results revealed that initial respiratory symptoms including cough (OR = 1.4, 95% CI: 1.2–1.7; *p* < 0.001), dyspnea (OR = 6.2, 95% CI: 3.6–10.6; *p* < 0.001), expectoration (OR = 1.7, 95% CI: 1.2–2.6; *p* < 0.01), and hemoptysis (OR = 4.0, 95% CI: 1.5–11.3; *p* < 0.001) occurred frequently in patients with severe COVID-19 pneumonia compared with the mild cases, while chest tightness (OR = 0.7, 95% CI: 0.2–2.4; *p* > 0.05) and pharyngalgia (OR = 1.2, 95% CI: 0.5–2.7; *p* > 0.05) did not show this characteristic.

### Associations Between Digestive Symptoms and Severe Cases

**Figure 7:**
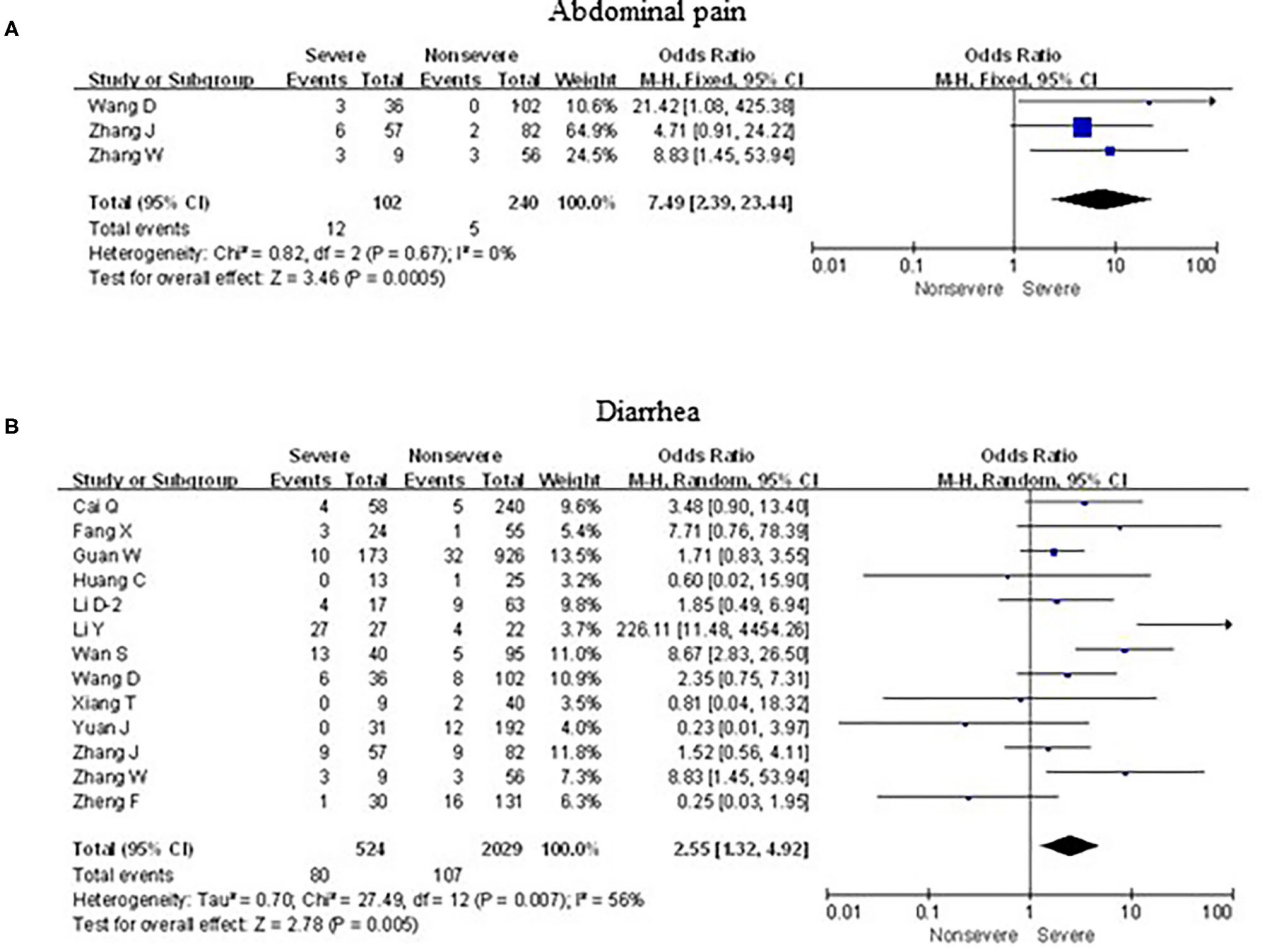
Forest plots of odds ratios (ORs) showed the associations between digestive symptoms, including abdominal pain **(A)** as well as diarrhea **(B)**, and disease progression of COVID-19 pneumonia.

As shown in Figure 7, meta-analysis results revealed that initial digestive symptoms including abdominal pain (OR = 7.5, 95% confidence interval [CI]: 2.4–23.4; *p* < 0.001) and diarrhea (OR = 2.6, 95% CI: 1.3–4.9; *p* < 0.001) occurred frequently in patients with severe COVID-19 pneumonia compared with the mild cases, while nausea or vomiting (OR = 0.9, 95% CI: 0.6–1.5; *p* > 0.05) did not show this characteristic.

### Associations Between Neurological Symptoms and Severe Cases

**Figure 8:**
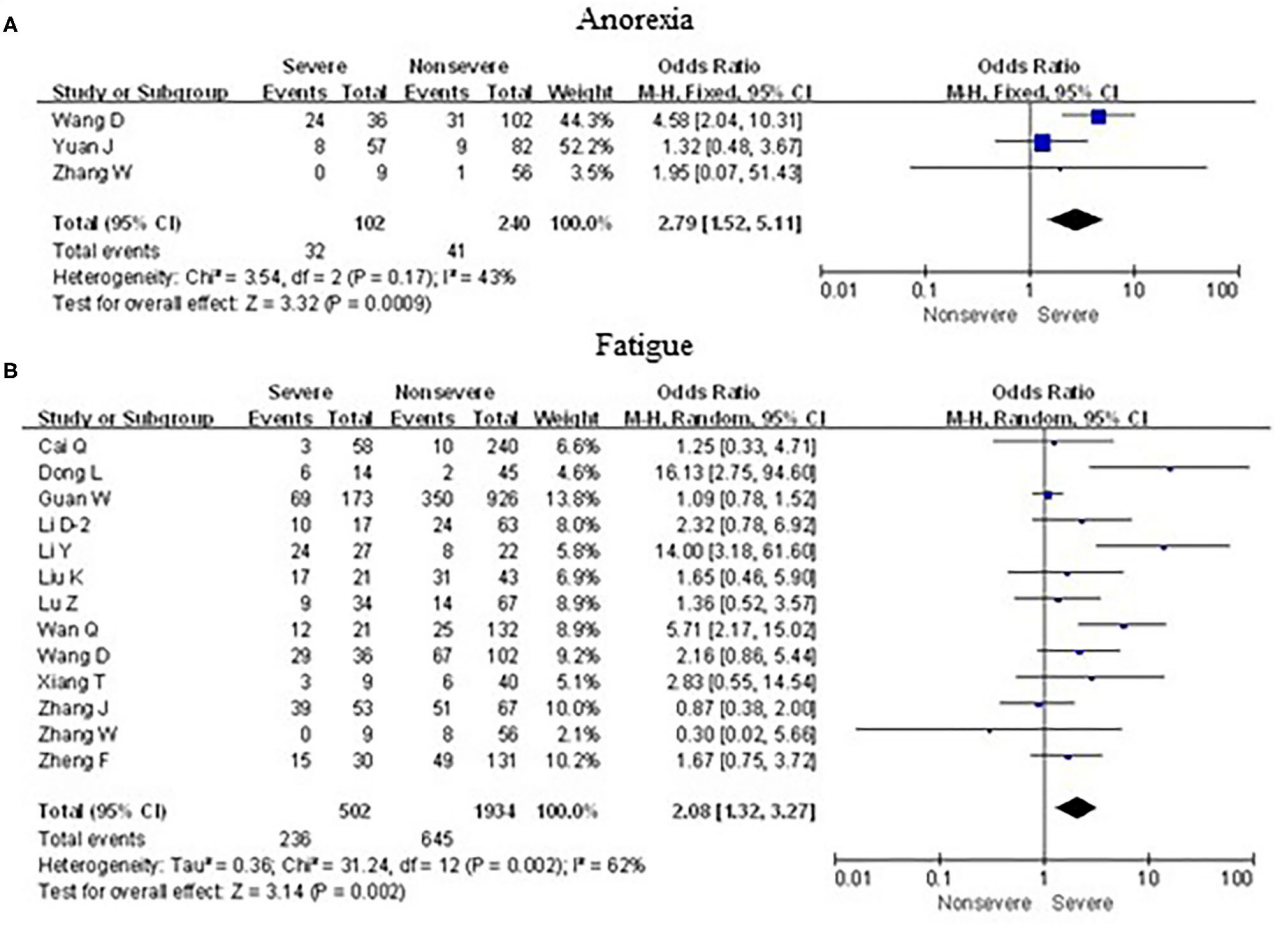
Forest plots of odds ratios (ORs) showed the associations between neurological symptoms, including anorexia **(A)** as well as fatigue **(B)**, and disease progression of COVID-19 pneumonia.

As shown in Figure 8, meta-analysis results revealed that initial neurological symptoms including anorexia (OR = 2.8, 95% CI: 1.5–5.1; *p* < 0.001) and fatigue (OR = 2.1, 95% CI: 1.3–3.3; *p* < 0.01) occurred frequently in patients with severe COVID-19 pneumonia compared with the mild cases, while headache (OR = 1.1, 95% CI: 0.8–1.6; *p* > 0.05) and myalgia (OR = 1.6, 95% CI: 0.8–3.1; *p* > 0.05) did not show this characteristic.

### Publication Bias

We used Begg's rank correlation test and Egger's weighted regression method to assess publication bias statistically. As shown in Figures 2A,B, neither Begg's (*p* = 0.453) nor Egger's (*p* = 0.246) test provided clear evidence of publication bias. These results showed the credibility of the findings reported in this meta-analysis.

## Discussion

Over the past month, nearly one million cases of coronavirus disease 2019 were confirmed in China and other countries in Asia, Africa, Europe, and America. COVID-19 has become an enormous threat to human health around the world and seriously hindered the development of the world economy and the progress of humanity. As a member of the Coronaviridae family, which is distributed widely in humans and other mammals such as bats, masked palm civets, and pangolins (16, 17), the novel coronavirus 2019 has a lower mortality but higher morbidity than MERS-CoV and SARS-CoV (18, 19). The novel coronavirus 2019 has been designated a Public Health Emergency of International Concern by the WHO because of its high infectivity (20). According to clinical symptoms, laboratory indicators, and imaging findings, COVID-19 is classified as mild, normal, severe, and critical (7). The diagnostic criteria for severe are any of the following: shortness of breath, respiratory rate (RR) > 30 times/min; oxygen saturation < 93% at rest; arterial blood oxygen partial pressure (PaO_2_)/oxygen concentration (FiO_2_) < 300 mmHg (1 mmHg = 0.133 kPa) (when the altitude is above 1,000 m, PaO_2_/FiO_2_ should be corrected according to the following formula: PaO_2_/FiO_2_ × [atmospheric pressure (mmHg)/760]). Pulmonary imaging showed that the lesions significantly progressed within 24–48 h, and >50% were managed according to severity (21). According to the analysis of existing clinical characteristics, severe patients tend to have dyspnea after 1 week in some cases, moderate to low fever, or even no obvious fever. Severe cases were more likely to rapidly develop to ARDS, septic shock, metabolic acidosis, and coagulopathy that are difficult to correct compared with non-severe patients. Besides, kidney, heart, and other organ damage, and even multiple organ failures were more likely to occur in severe patients (22, 23). It is necessary to predict and diagnose severe COVID-19 correctly as early as possible. Especially for countries and regions with poor medical conditions, we can make a preliminary classification of the severity according to the patient's signs, thereby reducing unnecessary medical waste and making the best use of medicines.

According to a statistical description of 656 and 1,994 patients with new coronary pneumonia counted by Alfonso J. Rodriguez-Morales and Long-Quan Li, the most common clinical symptoms of COVID-19 were fever, cough, and dyspnea (24, 25). Many similar studies have proved this conclusion. However, few studies have discussed how to initially assess the severity of patients with new coronary pneumonia in a short period, to facilitate earlier treatments and reduce complications and mortality. This meta-analysis involved the latest studies from March 2019 to April 2019 to analyze the clinical characteristics of the novel coronavirus 2019. Our study, which included 3,326 confirmed cases, provided more credible results of clinical features of the novel coronavirus 2019. Although all the studies were case studies, there was no publication bias. In this study, the dominant clinical features of COVID-19 patients were cough (98.5%) and fever (93.4%), which were similar to other studies on initial symptoms of COVID-19 patients in China (22, 26, 27). By analyzing the distribution differences of initial symptoms between severe cases and mild cases, we found that some initial symptoms including abdominal pain (OR = 7.5, 95% CI: 2.4–23.4), dyspnea (OR = 6.2, 95% CI: 3.6–10.6), hemoptysis (OR = 4.0, 95% CI: 1.5–11.3), anorexia (OR = 2.8, 95% CI: 1.5–5.1), diarrhea (OR = 2.6, 95% CI: 1.3–4.9), fatigue (OR = 2.1, 95% CI: 1.3–3.3), expectoration (OR = 1.7, 95% CI: 1.2–2.6), fever (OR = 1.5, 95% CI: 1.2–1.9), and cough (OR = 1.4, 95% CI: 1.2–1.7) occurred more frequently in severe COVID-19 patients than in mild COVID-19 patients. However, chest tightness (*p* = 0.55), headache (*p* = 0.48), myalgia (*p* = 0.15), nausea or vomiting (*p* = 0.66), and pharyngalgia (*p* = 0.69) did not show a significant difference between patients with severe and mild COVID-19. In other words, the symptoms, including abdominal pain, anorexia, diarrhea, dyspnea, expectoration, fatigue, cough, fever, and hemoptysis, had a different distribution in severe cases and mild cases of coronavirus disease 2019, especially abdominal pain, dyspnea, and hemoptysis. The outcomes of our study were of great significance in predicting and diagnosing severe 2019 coronavirus disease, reducing the mortality and preventing its further spread. Some countries with severe epidemics do not have sufficient medical resources such as Italy, Spain, and India. They should pay more attention to the COVID-19 patients whose initial symptoms are abdominal pain, dyspnea, hemoptysis, anorexia, diarrhea, or fatigue because these patients are more likely to deteriorate to a severe type of 2019 coronavirus disease. Moreover, to further reduce the mortality of patients, medical workers should monitor their vital signs closely.

Severe acute respiratory syndrome (SARS) is an infectious disease belonging to the coronavirus family that occurred in 2002, and it has very similar initial symptoms and complications as the new coronavirus (1). According to research published in *The Lancet Infectious Diseases* in 2004, symptoms such as fever, cough, and respiratory distress could serve as prediction and monitoring indicators for critical SARS (28). The result is consistent with our research. Moreover, Daozheng Huang et al. summarized 4,972 patients with COVID-19 from the end of 2019 to February 12, 2020 (29). They found that some clinical symptoms such as dyspnea, vomiting, and diarrhea had distinct differences between severe and non-severe patients, but no apparent differences were found in the symptoms of headache, fever, myalgia, and arthralgia. Also, calcitonin ≥ 0.05 ng/mL, creatinine > 104 μmol/L, lymphocyte count < 1.5 ×10^9^/L, and bilateral involvement of chest CT were significant risk factors for severe COVID-19. The above findings are highly consistent with our results, while vomiting and fever are contrary to our results. Therefore, it is necessary to expand the sample size of the research subjects for further exploration. What is certain is that when patients show abdominal pain, anorexia, diarrhea, dyspnea, fatigue, cough, or hemoptysis, they are more likely to be diagnosed as severe cases in the later development of the disease.

Our study still has some limitations. First, all the studies included were from China, so it would be better to involve studies in other countries to get a more comprehensive result of COVID-19, and the outcomes might be affected by geographical and ethnic differences. Second, more detailed patient information such as laboratory findings and ages were not involved in our meta-analysis for lack of relevant data. Third, some outcomes (including hemoptysis, abdominal pain, and anorexia) were defined with only three studies since many articles have not been published yet, which might increase the risk of bias. Finally, we performed this meta-analysis during the ongoing outbreak of COVID-19, and many regions affected by COVID-19 have not yet presented clinical data, which might skew the results of the analysis.

In conclusion, our study analyzed 3,326 cases and found that some initial symptoms especially abdominal pain, dyspnea, hemoptysis, anorexia, diarrhea, and fatigue were significantly correlated with a severe type of 2019 coronavirus disease, suggesting that these symptoms could predict severe novel coronary pneumonia. Hospitalized COVID-19 patients with these initial symptoms should be paid more attention to keep a stable vital sign.

## Data Availability Statement

The original contributions presented in the study are included in the article/supplementary materials, further inquiries can be directed to the corresponding author/s.

## Author Contributions

XH: data statistics and analysis. XC: draft writing. XF: literature screening. SC: language modification. HW and MX: paper modification. All authors contributed to the article and approved the submitted version.

## Conflict of Interest

The authors declare that the research was conducted in the absence of any commercial or financial relationships that could be construed as a potential conflict of interest.
